# Decreased Brain K_ATP_ Channel Contributes to Exacerbating Ischemic Brain Injury and the Failure of Neuroprotection by Sevoflurane Post-Conditioning in Diabetic Rats

**DOI:** 10.1371/journal.pone.0073334

**Published:** 2013-08-26

**Authors:** Dongliang Li, Bin Huang, Jiangdong Liu, Liang Li, Xingang Li

**Affiliations:** 1 Department of Anesthesiology, Qilu Hospital, Shandong University, Jinan City, Shandong Province, China; 2 Department of Neurosurgery, Qilu Hospital, Shandong University, Jinan City, Shandong Province, China; 3 Brain Science Research Institute, Shandong University, Jinan City, Shandong Province, China; 4 Department of Anesthesiology, The people's hospital of Gaomi, Gaomi City, Shandong Province, China; Massachusetts General Hospital/Harvard Medical School, United States of America

## Abstract

Diabetes leads to exacerbating brain injury after ischemic stroke, but the underlying mechanisms and whether therapeutic intervention with anesthetic post-conditioning can induce neuroprotection in this population are not known. We tested the hypothesis that alteration of brain mitochondrial (mito) K_ATP_ channels might cause exacerbating brain injury after ischemic stroke and attenuate anesthetic post-conditioning induced neuroprotection in diabetes. We also examined whether hyperglycemic correction with insulin would restore anesthetic post-conditioning in diabetes. Non-diabetic rats and diabetic rats treated with or without insulin were subjected to focal cerebral ischemia for 2 h followed by 24 h of reperfusion. Post-conditioning was performed by exposure to sevoflurane for 15 min, immediately at the onset of reperfusion. The role of the mitoK_ATP_ channel was assessed by administration of a selective blocker 5-hydroxydecanoate (5-HD) before sevoflurane post-conditioning or by diazoxide (DZX), a mitoK_ATP_ channel opener, given in place of sevoflurane. Compared with non-diabetic rats, diabetic rats had larger infarct volume and worse neurological outcome at 24 h after ischemia. Sevoflurane or DZX reduced the infarct volume and improved neurological outcome in non-diabetic rats but not in diabetic rats, and the protective effects of sevoflurane in non-diabetic rats were inhibited by pretreatment with 5-HD. Molecular studies revealed that expression of Kir6.2, an important mitoK_ATP_ channel component, was decreased in the brain of diabetic rats as compared to non-diabetic rats. In contrast, hyperglycemic correction with insulin in diabetic rats normalized expression of brain Kir6.2, reduced ischemic brain damage and restored neuroprotective effects of sevoflurane post-conditioning. Our findings suggest that decreased brain mitoK_ATP_ channel contributes to exacerbating ischemic brain injury and the failure of neuroprotection by anesthetic post-conditioning in diabetes. Insulin glycemic control in diabetes may restore the neuroprotective effects of anesthetic post-conditioning by modulation of brain mitoK_ATP_ channel.

## Introduction

Diabetes is a devastating disease of epidemic proportions. It is estimated that more than 220 million patients are affected by diabetes worldwide [1,2]. Epidemiological studies have suggested that diabetes is a critical risk factor for ischemic stroke, which is one of the leading causes of death and permanent disability in humans [3,4]. In addition, diabetes complicates ischemic injury, leading to increased neuronal damage and poor functional recovery [5,6]. Therefore, exploring the mechanisms underlying ischemic brain injury under diabetic conditions and develop more effective therapies for neuroprotection in this population has been a major focus of medical research in recent years. However, very few treatments to reduce ischemic brain injury in clinical practice have been established.

A steadily increasing number of investigations demonstrate that pre- or post-conditioning with volatile anesthetics attenuates ischemia-induced brain injury and the mechanisms are thought to be similar to ischemic pre- or post-conditioning [7]. Although ischemic or anesthetic pre-conditioning is an effective process for protection against ischemic brain injury, its clinical use is limited as ischemic episodes are mostly unpredictable. However, the onset of reperfusion is more often predictable [8]. Therefore, the performance of post-conditioning, through modulation of reperfusion rather than ischemia, is a more clinically feasible option. The clinical application of ischemic post-conditioning to reduce neuronal damage is also limited by the fact that no optimal maneuvers of repeated cycles of brief reperfusion and reocclusion have been devised, and such maneuvers may be dangerous to some patients. Instead, anesthetic post-conditioning induced by anesthetic application started immediately after ischemia or during early reperfusion may offer appropriate therapeutic potential for neuronal protection against ischemia-induced brain injury. To date, the majority of studies investigating neuroprotective effects of anesthetic post-conditioning have been conducted in healthy animals, and there is no study that has addressed the effects of anesthetic post-conditioning in the presence of hyperglycemia or diabetes. It is therefore unknown whether diabetes influences anesthetic-induced neuroprotection by post-conditioning.

It has been well established that mitochondrial K_ATP_ (mitoK_ATP_) channels play an important role in protecting neurons against ischemic damage [9]. Recent studies demonstrated that mitoK_ATP_ channels mediate neuroprotective effects of anesthetic sevoflurane post-conditioning in a rat model of focal cerebral ischemia [8,10]. However, expression or function of mitoK_ATP_ channels has been found to be impaired in the brain and peripheral tissues in the presence of hyperglycemia or diabetes [11,12].

Accordingly, the aims of the current study were to test the hypothesis that alteration of brain mitoK_ATP_ channels in diabetes might cause exacerbating brain injury following ischemic stroke and attenuate anesthetic sevoflurane post-conditioning induced neuroprotection. In addition, we examined whether hyperglycemic correction with insulin would restore sevoflurane post-conditioning in diabetes.

## Methods

### Induction of diabetic Animals

Male Sprague-Dawley rats weighing 125-140 g were purchased from Beijing Laboratory Animal Research Center (Beijing, China) and housed under standard laboratory conditions; water and Purina 5010 rodent chow were continuously available. All experiments were approved by the Institutional Animal Care and Use Committee at the Shandong University and were performed in accordance with the “Guiding Principles for Research Involving Animals and Human Beings”.

Diabetic (DB) rats were induced as previously described [13]. Briefly, an intraperitoneal injection of 40 mg/kg streptozotocin (STZ, Sigma, St. Louis, MO) in ice-cold 0.5 mol/l citrate buffer (pH 4.5) was administered. A second dose of STZ (40 mg/kg) was injected 24 h later. Blood glucose levels were monitored from the tail vein using a glucose analyzer (Prestige Smart System) immediately before receiving STZ and 3 days after injection of STZ, and then every week after STZ administration for 8 weeks. An animal with a blood glucose level of >300 mg/dl at 3 days after injection of STZ was considered to be diabetic. Rats receiving vehicle injection (citrate buffer) served as the non-diabetic (Non-DB) group.

### Experimental protocol

Non-DB and DB rats were randomly assigned to 10 groups (n=8 for each group) as follows:

(1) Non-DB ischemia-reperfusion alone group (Non-DB-Con)

(2) Non-DB sevoflurane post-conditioning group (Non-DB-Sev)

(3) Non-DB sevoflurane post-conditioning plus 5-hydroxydecanoate (5HD) group (Non-DB-5HD+Sev). Same as group (2), but treated with 5HD, a selective mitoK_ATP_ channel blocker (40 mg/kg i.p.), 30 min before sevoflurane post-conditioning [8]

(4) Non-DB diazoxide (DZX) alone group (Non-DB-DZX). Rats were treated with DZX, a mitoK_ATP_ channel opener (10 mg/kg i.p.), 30 min before reperfusion [8]

(5) DB ischemia-reperfusion alone group (DB-Con)

(6) DB sevoflurane post-conditioning group (DB-Sev)

(7) DB sevoflurane post-conditioning plus 5-hydroxydecanoate (5HD) group (DB-5HD+Sev)

(8) DB diazoxide (DZX) alone group (DB-DZX)

(9) Insulin-treated DB ischemia-reperfusion alone group (Ins-DB-Con). Same as group (5), but these DB rats received subcutaneous insulin implants (Linplants; LinShin Canada, Scarborough, Toronto, ON, Canada) for 4 weeks, prior to ischemia-reperfusion. The implants were 7 mm in length and 2 mm in diameter, and they released insulin (in microrecrystalized palmitic acid) at 2 units/24 h

(10) Insulin-treated DB sevoflurane post-conditioning group (Ins-DB-Sev). Same as group (6), but these DB rats received subcutaneous insulin implants for 4 weeks, prior to ischemia-reperfusion

Eight weeks after STZ injection, these rats were prepared for ischemic-reperfusion study. After a 30 min stabilization period, all rats were subjected to 2 h of focal cerebral ischemia followed by 24 h reperfusion. Rats assigned to sevoflurane post-conditioning were placed in the gas-tight anaesthesia chamber and exposed for 15 min to sevoflurane at a concentration of 2.6% immediately at the onset of reperfusion. A gas analyser was connected to the chamber to monitor and maintain the concentrations of inspired oxygen and sevoflurane constantly. The dose for sevoflurane was chosen according to a previous study in which this dose was determined to result in optimal neuroprotective actions in vivo in rats [8]. At the end of the protocol (24 h after ischemia), neurological deficit scores and motor coordination were evaluated. Rats were then sacrificed, the blood samples were collected for biochemical measurements and the brains were removed for infarct volume assessment.

### Transient focal cerebral ischemia

Transient focal cerebral ischaemia was induced by middle cerebral artery occlusion with subsequent reperfusion as previously described [6]. Briefly, rats were anesthetized with an intraperitoneal (i.p.) injection of chloral hydrate (300 mg kg^−1^). Body core temperature was maintained within a normothermic range (37°C to 38°C) with a temperature-controlled heating pad. The right common carotid artery, the external carotid artery and the internal carotid artery were isolated through a midline cervical incision. A 4/0 surgical nylon monofilament was introduced into the right common carotid artery and advanced until faint resistance was felt at approximately 18.0 ± 0.5 mm from the carotid bifurcation. After 2 h of occlusion, the monofilament was removed to allow reperfusion for 24h.

The right femoral artery was also cannulated for monitoring blood pressure (BP) and heart rate (HR) and for arterial blood gas measurements. Aortic pressure signals were digitized using an analog-digital converter and recorded on a computer using the PowerLab software (PowerLab/8SP, Chart 5.0; ADInstruments Pty, Ltd., Castle Hill, Australia). BP and HR measurements were performed immediately before ischemia, 15 and 35 min after the onset of ischemia, and 5 and 15 min after the onset of reperfusion. Arterial blood gas measurements were performed 15 min after the onset of ischemia or reperfusion using a blood gas analyzer (Compact 3, AVL Medizintechnik). After recovery from anesthesia, the animals were transferred to their cages and were allowed free access to food and water.

### Evaluation of neurological deficit scores, motor coordination and infarct volume

Neurological deficit scores were evaluated 24 h after ischemia based on an eight-point scale [8,14]. The score was 0 for no apparent deficits; 1 for failure to extend left forepaw fully; 2 for decreased grip of the left forelimb; 3 for spontaneous movement in all directions, contralateral circling only if pulled by the tail; 4 for circling or walking to the left; 5 for walking only if stimulated; 6 for unresponsiveness to stimulation and with depressed level of consciousness; and 7 for death.

Motor coordination was evaluated 24 h before and after ischemia, respectively. The experimental procedure was described previously [15]. Briefly, the duration (s) that the rats stayed on a rotating rod was recorded automatically in each case for up to 180 s. The trial was conducted five times for each rat, and the mean riding time was used as the mean value for this test. When the duration of riding was over 180 s, the rat was released from the rod, and the riding time was recorded as 180 s.

At the end of the observation period, rats were euthanized and brains were quickly removed for infarct volume assessment, as previously described [6]. Briefly, brains were sectioned at 2-mm intervals throughout the rostrocaudal axis of the striatum. Slices were then staining with 2% 2,3,5 triphenyltetrazolium chloride (TTC) for 15 min at 37^°^C. Slice images were digitalized and infarct areas were quantified using NIH Image 1.60. The Complete lack of staining with TTC was defined as the infarct lesion. The infarct volume was expressed as a percentage of the contralateral hemisphere.

### Biochemical measurement

Plasma insulin level was measured by commercial ELISA kits (Cayman, Michigan, USA).

### Analysis of mitoK_ATP_ channel mRNA

Eight weeks after STZ injection, additional non-DB rats and DB rats that were treated with or without insulin for 4 weeks but not subjected to ischemia-reperfusion (n=13 for each group), were used for quantitative analysis of brain mitoK_ATP_ channel expression or for immunofluorescent study.

mRNA expression of mitoK_ATP_ channel subunits was measured by RT-PCR. The brain tissue was homogenized in TRIzol reagent (Invitrogen, Carlsbad CA) using a Polytron homogenizer. Total RNA was extracted and reverse transcribed into cDNA using 10× buffer (MgCl_2_ free; PerkinElmer), 10 mM dNTPs, 25 mM MgCl_2_, random hexamer primers, RNasin (33 U/µl, Promega), and Maloney murine leukemia virus reverse transcriptase (10 U/µl, Promega). The reaction mixture was incubated at 21°C for 10 min and held at 42°C for 75 min followed by 5 min at 95°C. Five microliters of each RT reaction were used for PCR reactions for the mitoK_ATP_ channel subunits Kir6.2 and SUR1. PCR was performed in total 50 µl using specific primers for Kir6.2 (forward 5'-CGCATGGTGACAGAGGAATG-3’, reverse 5'-GTGGAGAGGCACAACTTCGC-3’), SUR1 (forward 5'-TGCCAGCTCTTTGAGCATTG-3’, reverse 5'-AGGATGATACGGTTGAGCAGG-3’), GAPDH (forward 5'-CTCAAGATTGTCAGCAATGC-3’, 5'-CAGGATGCCCTTTAGTGGGC-3’) and Taq DNA polymerase (Roche). The 35 cycles included initial denaturation at 94°C, 4 min; 94°C, 10 sec; 56°C, 30 sec; 72°C, 2 min; and final extension 72°C, 10 min. PCR products (12 µl) were separated by electrophoresis on a 1.5% agarose gel stained with ethidium bromide and visualized with a Molecular Imager (Bio-Rad, Hercules, CA).

### Analysis of mitoK_ATP_ channel protein

Brain mitochondria were isolated using a previously described method [16]. Briefly, Brain was homogenized in ice-cold isolation buffer containing (in mmol/l) 225 mannitol, 75 sucrose, 5 MOPS, 0.5 EGTA, and 2 taurine, with 0.2% BSA (pH 7.25). The homogenate was centrifuged twice at 1,000 *g* for 5 min (4°C), and the supernatant was then centrifuged at 10,000 *g* for 10 min (4°C). After the pellet was washed, it was resuspended in buffer containing (in mmol/l) 225 mannitol, 25 sucrose, 5 MOPS, 1 EGTA, 5 KH_2_PO_4_, and 2 taurine supplemented with 0.2% BSA (pH 7.4), placed on ice, and used within 3 h. The concentration of mitochondrial protein was determined by the Bradford method. Equal amounts of protein from mitochondrial lysate samples were separated by 4–20% SDS-PAGE, transferred onto a PVDF membrane, and blocked with 5% skim milk, Tris-buffered saline, and 0.1% Tween 20. Blots were incubated with rabbit polyclonal anti-Kir6.2, rabbit polyclonal anti-SUR1 (EMD Millipore) and rabbit polyclonal anti β-Actin (Santa Cruz). The membranes were then washed and incubated with goat-anti rabbit HRP-IgG (Santa Cruz). The bound antibodies were visualized by enhanced chemiluminescence, and the densities of the immunobands were quantitated.

### Immunofluorescent study

Three rats from each group were perfused transcardially with heparinized saline followed by ice-cold 4% paraformaldehyde. Brains were removed and fixed overnight in 4% paraformaldehyde at 4°C and then immersed in 30% sucrose. Brain tissue was sliced into 16-µm coronal sections. The sections were incubated with the primary antibodies, the rabbit polyclonal antibody to Kir6.2 and the mouse monoclonal antibody to SUR1 (Millipore) followed by secondary antibodies Alex Fluor 488 goat anti-rabbit IgG and Alex Fluor 568 goat anti-mouse IgG (Invitrogen). Fluorescent intensity was quantified with NIH Image 1.60. In each rat, ≥5 representative tissue samples from brain cortex were examined using a confocal laser-scanning microscope (Zeiss LSM 510, Carl Zeiss, Inc), and an average value was reported.

### Statistical analysis

All data are expressed as mean ± S.E.M. Changes in blood pressure and heart rate between groups or between time points in a group were performed using two-way analysis of variance followed by Tukey’s post hoc test. Data for neurological deficit scores, motor coordination, infarct volumes, biochemical parameters and mitoK_ATP_ channel expression were analyzed by Student’s *t* test with Bonferroni’s correction for multiple comparisons. *P*<0.05 was considered statistically significant.

## Results

### Characteristics of diabetic animal model

Baseline body weight (175 ± 6 g), the levels of blood glucose (118 ± 5 mg/dl) and blood insulin (2.61 ± 0.22 ng/mL) were similar in all groups at the time of STZ injections. As shown in table **1**, eight weeks after STZ injections, the diabetic rats displayed weight loss of approximately 38% compared with non-diabetic rats (*P* < 0.01). At this time point, the levels of blood glucose and blood insulin remained the same in all non-diabetic rats, however, the blood glucose levels were significantly higher and blood insulin levels were significantly lower in diabetic rats than non-diabetic rats. Insulin treatment prevented the diabetes-induced body weight decrease and blocked the blood glucose increase. In these rats receiving insulin treatment, insulin levels were significantly higher at 8 weeks of diabetes than those in the untreated diabetic rats. Two diabetic rats died during diabetes induction, three non-diabetic and 4 diabetic rats died during ischemia-reperfusion.

**Table 1:** Characteristics of non-diabetic and diabetic rats at termination of the study.

Group	Body weight (g)	Blood glucose (mg/dl)	Blood insulin (ng/ml)
**Non-DB-Con**	485 ± 19	122 ± 5	2.80 ± 0.19
**Non-DB-Sev**	472 ± 15	119 ± 4	2.72 ± 0.25
**Non-DB-5HD+Sev**	483 ± 20	120 ± 5	2.74 ± 0.31
**Non-DB-DZX**	479 ± 17	125 ± 3	2.69 ± 0.27
**DB-Con**	352 ± 16*	522 ± 13*	0.25 ± 0.03*
**DB-Sev**	340 ± 13*	510 ± 12*	0.20 ± 0.02*
**DB-5HD+Sev**	348 ± 15*	509 ± 15*	0.19 ± 0.02*
**DB-DZX**	353 ± 12*	513 ±17*	0.23 ± 0.04*
**Ins-DB-Con**	450 ± 21†	128 ± 9†	4.16 ± 0.35*†
**Ins-DB-Sev**	445 ± 22†	134 ± 10†	4.07 ± 0.32*†

Values are mean ± SEM; n = 6-8 for each group. *P<0.05 vs. Non-DB-Con; †*P*<0.05 vs. DB-Con.

### Hemodynamic and physiological variables

Hemodynamic and physiological variables, including BP, HR and arterial blood gases, were monitored and controlled before, during and after focal cerebral ischemia to reduce confounding factors on neurological outcomes. There were no significant differences among groups in mean BP, HR, arterial pH, carbon dioxide tension (Pco_2_) and arterial oxygen tension (Po_2_) at each time point before, during focal cerebral ischemia and during reperfusion (data not shown).

### Effects of sevoflurane post-conditioning or DZX on neuronal injury

**Figure 1:**
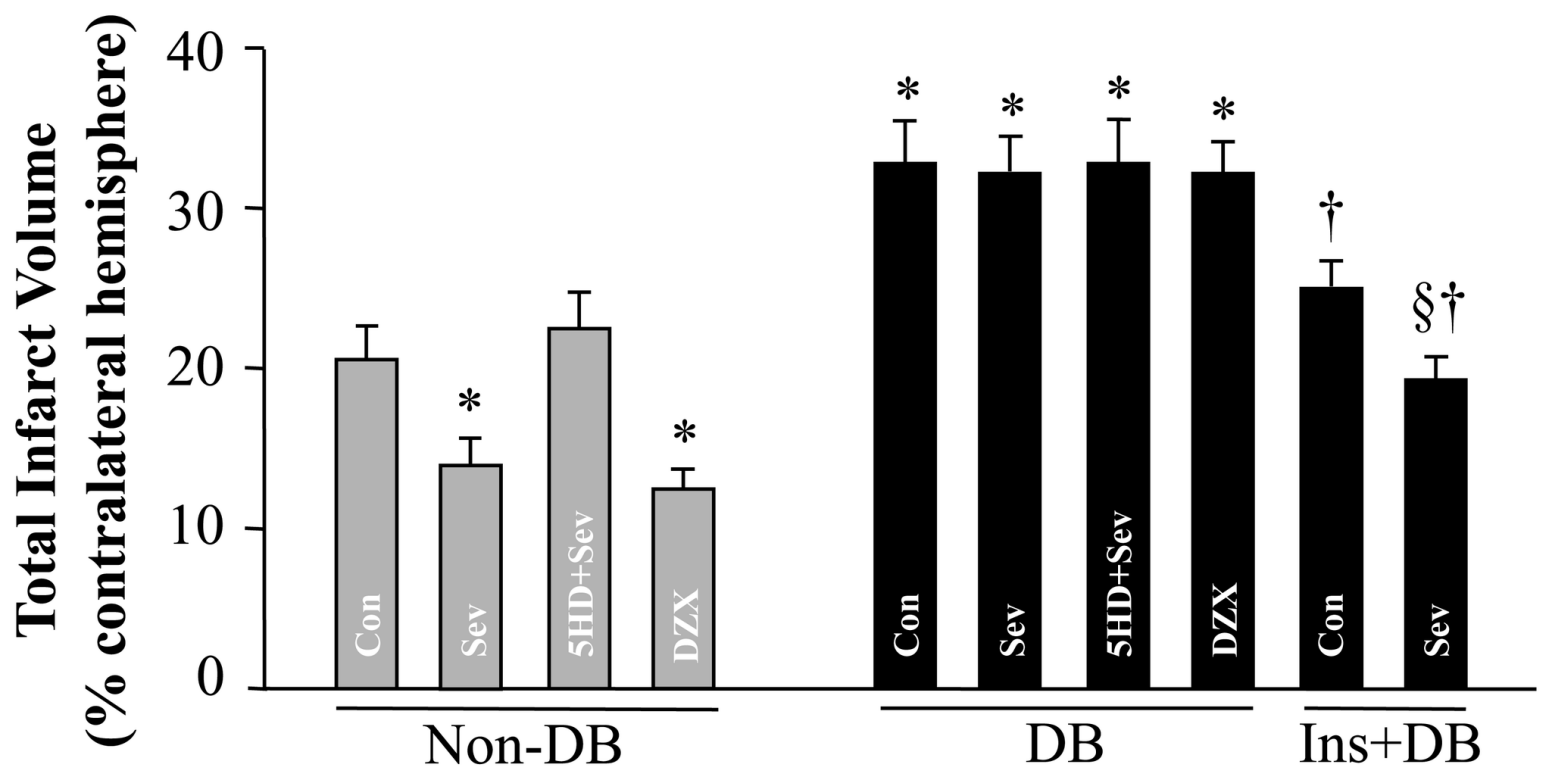
Infarct volume 24 h after middle cerebral artery occlusion and reperfusion in each group. Infarction volume was determined as a percentage of the contralateral hemisphere. Values were expressed as mean ± SEM (n=6-8 for each group). *P<0.05 vs. Non-DB-Con or Non-DB-5HD+Sev; † *P*<0.05, Ins-DB-Con or Ins-DB-Sev vs. DB-Con, §*P*<0.05, Ins-DB-Con vs. Ins-DB-Sev.

Total infarct volume induced by a 2-h ischemia followed by 24 h of reperfusion was significantly greater in the diabetic control rats as compared to the non-diabetic control rats (32 ± 3% * vs. 21 ± 2%, DB-Con vs. Non-BD-Con, *P < 0.05) (Figure **1**). Sevoflurane post-conditioning or mitoK_ATP_ channel opener DZX similarly reduced infarct volume in non-diabetic rats, whereas both failed to protect diabetic rats against ischemic cerebral injury. In addition, administration of mitoK_ATP_ channel blocker 5-HD before sevoflurane post-conditioning completely inhibited the protective effect of sevoflurane postconditioning in non-diabetic rats, whereas it had no effect (versus DB-Con, *P* = not significant) in diabetic rats.

**Figure 2:**
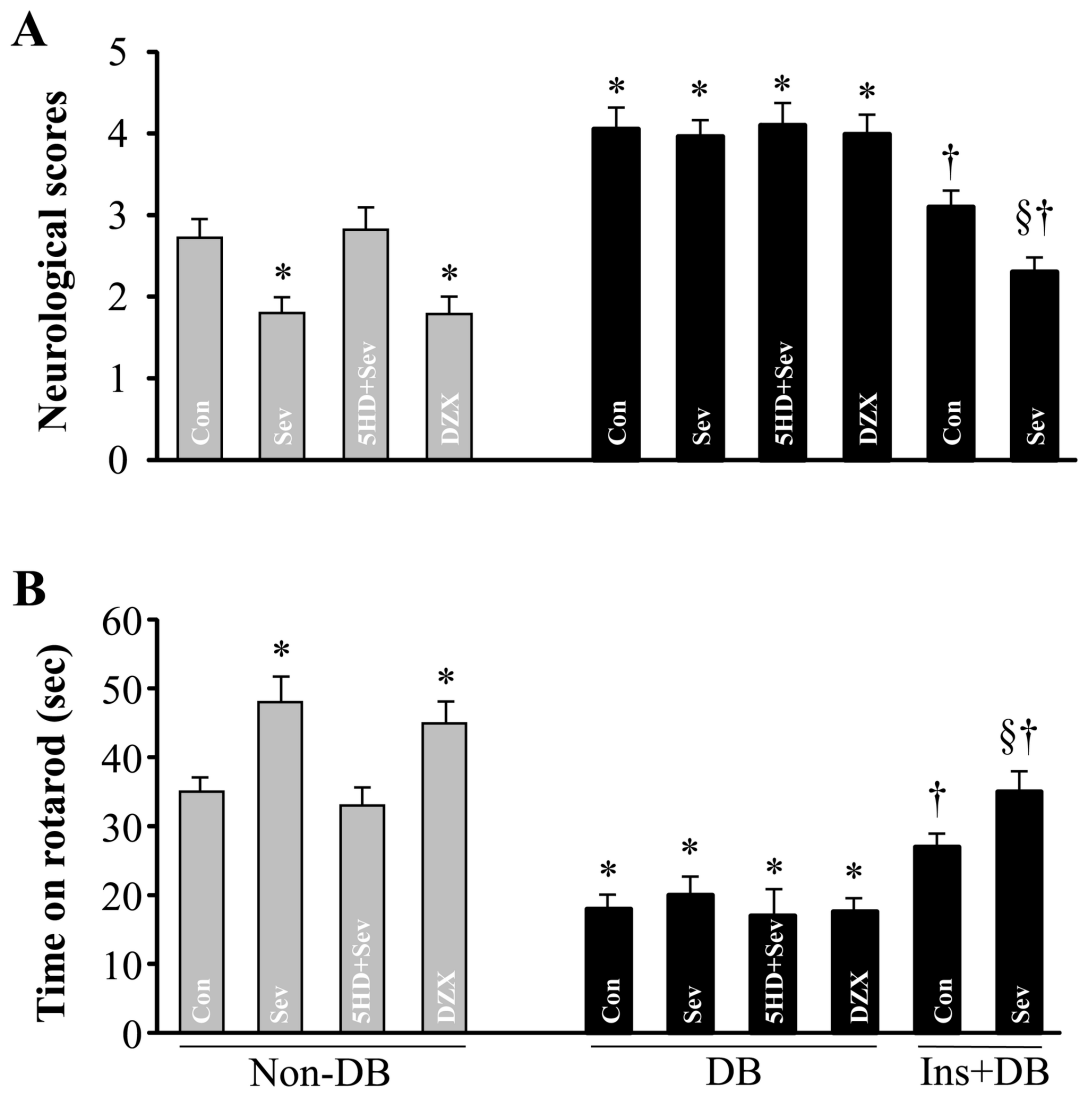
Neurological deficit scores (A) and motor coordination (B) 24 h after middle cerebral artery occlusion and reperfusion in each group. Values were expressed as mean ± SEM (n=6-8 for each group). *P<0.05 vs. Non-DB-Con or Non-DB-5HD+Sev; † *P*<0.05, Ins-DB-Con or Ins-DB-Sev vs. DB-Con, §*P*<0.05, Ins-DB-Con vs. Ins-DB-Sev.

There was no difference of time in rotarod among groups before ischemia (average time in rotarod was 153 ± 11 sec). Results of neurological severity scores and rotarod performance 24 h after ischemia from each group are presented in Figure **2**. The neurological scores were higher (Figure **2A**) and the time in rotarod (Figure **2B**) was decreased after 24 h of reperfusion in diabetic control rats compared with non-diabetic control rats. Sevoflurane post-conditioning and DZX equally reduced neurological scores and improved rotarod performance (*P* < 0.05 vs. Non-BD-Con) in non-diabetic rats, but these beneficial effects induced by sevoflurane post-conditioning were completely reversed by pretreatment with 5-HD. In contrast, neither neurological scores nor rotarod performance were changed by sevoflurane post-conditioning, administration of DZX or 5-HD in diabetic rats (vs. DB-Con, *P* = not significant).

Diabetic rats receiving a 4-week insulin treatment exhibited significantly smaller infarct volume compared with untreated diabetic rats (24 ± 2% * vs. 32 ± 3%, Ins-DB-Con vs. BD-Con, *P < 0.05) (Figure **1**). In addition, insulin-treated diabetic rats also had lower neurological scores (Figure **2A**) and increased time in rotarod (Figure **2B**) than untreated diabetic rats. Furthermore, post-conditioning with sevoflurane in these rats further reduced infarct volume, decreased neurological scores and improved rotarod performance as compared to insulin-treated control rats.

### Expression of mitoK_ATP_ channels in the brain

**Figure 3:**
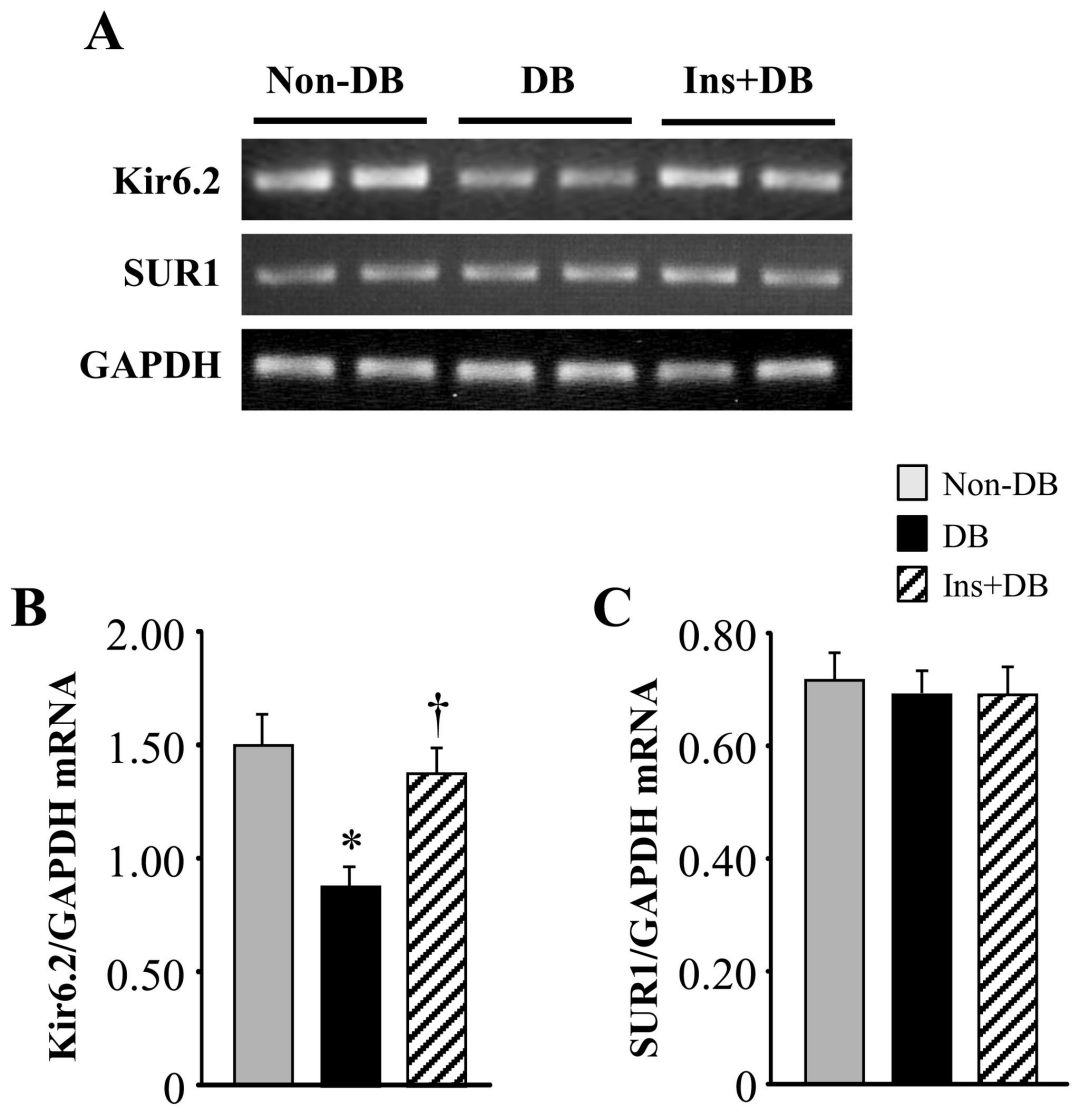
Representative RT-PCR product bands (A) and quantitative comparison of mRNA expression for brain mitoK_ATP_ channel subunits Kir6.2 (B) and SUR1 (C) from non-diabetic rats and diabetic rats treated with or without insulin. Values are expressed as means ± SEM (n=6 for each group). *P<0.05 vs. Non-DB; † *P*<0.05 vs. DB.

**Figure 4:**
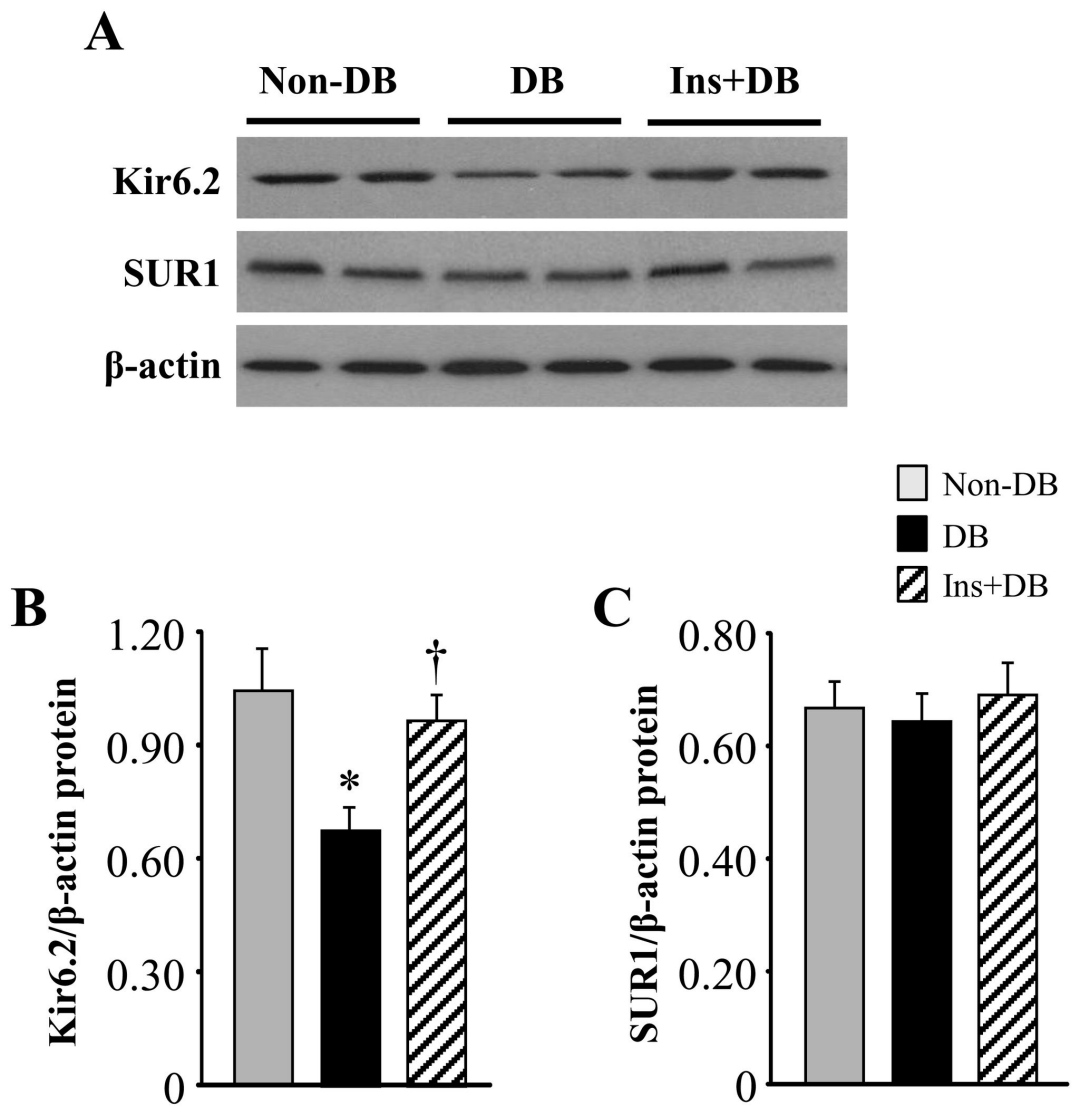
Representative Western blots (A) and quantitative comparison of protein levels for Kir6.2 (B) and SUR1 (C) from non-diabetic rats and diabetic rats treated with or without insulin. Values are expressed as means ± SEM (n=4 for each group). *P<0.05 vs. Non-DB; † *P*<0.05 vs. DB.

To determine whether diabetes alter brain mitoK_ATP_ channels, we measured the expression of mitoK_ATP_ channel subunits Kir6.2 and SUR1 in the brain. As shown in Figures **3** and **4**, real**-**time PCR and Western blot analyses revealed the presence of mRNA and protein of both Kir6.2 and SUR1 in the brain of non-diabetic and diabetic rats 8 weeks after STZ infusion. Kir6.2 mRNA (Figure **3**) and protein expression (Figure **4**) was significantly (*P* < 0.05) lower in the brain of diabetic rats compared with non-diabetic rats. However, there were no differences between two groups in SUR1 mRNA and protein expression in the brain. Four-week insulin treatment of diabetic rats normalized Kir6.2 mRNA and protein expression, but did not change SUR1 expression in the brain.

### K_ATP_ channels immunostaining in the brain

**Figure 5:**
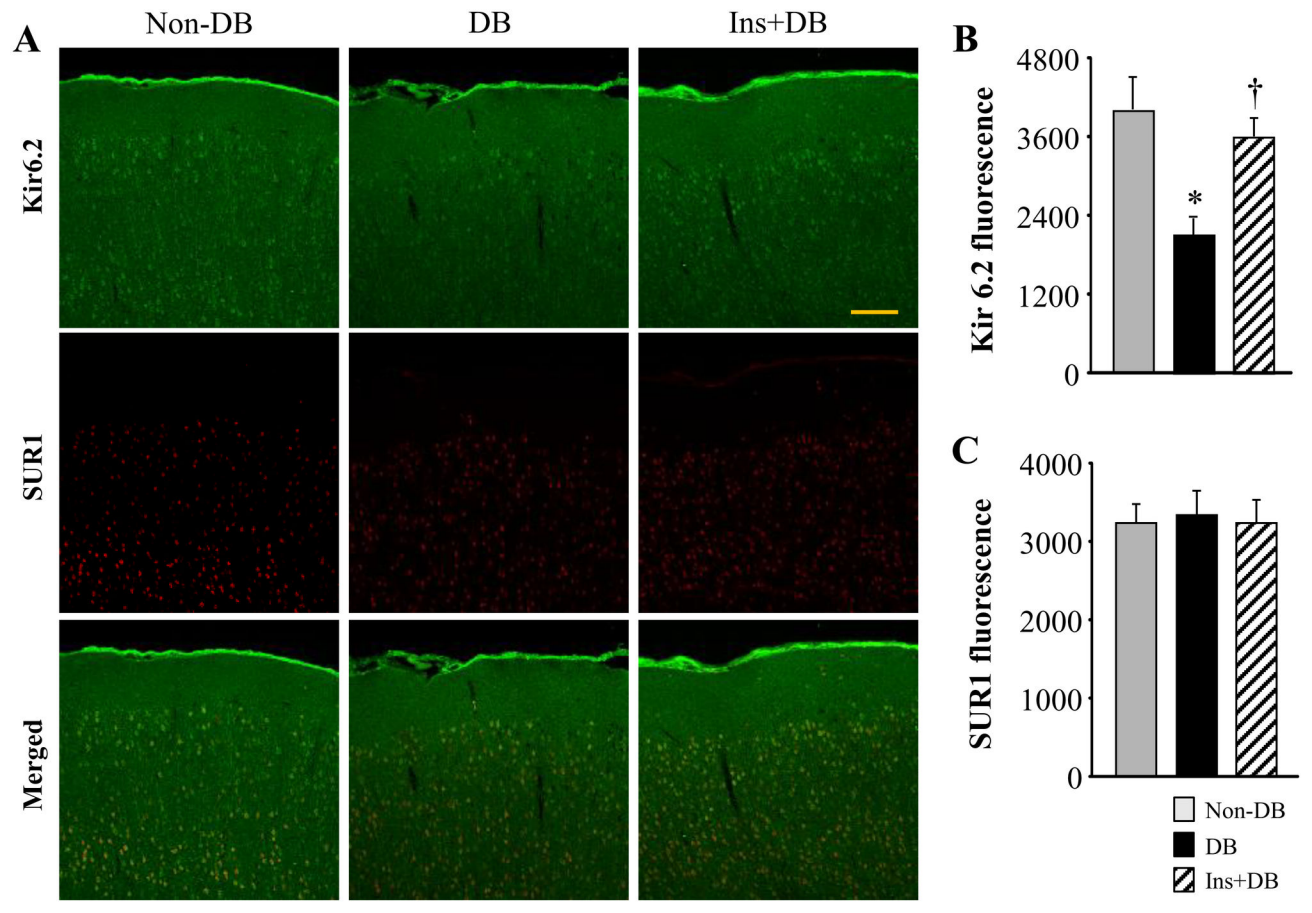
Representative laser confocal images (A) and Quantitative comparison of Kir6.2 (B) and SUR1 (C) fluorescence in the brain cortex from non-diabetic rats and diabetic rats treated with or without insulin. Scale bar, 200 µm. Values are expressed as means ± SEM (n=3 for each group). *P<0.05 vs. Non-DB; † *P*<0.05 vs. DB.

Laser confocal microscopy showed that Kir6.2 immunoreactivity in the brain cortex was significantly reduced in diabetic rats compared with non-diabetic rats (Figure **5**). After 4-week treatment with insulin, Kir6.2 immunoreactivity was increased in the brain of diabetic rats. In contrast, SUR1 immunoreactivity in the brain cortex was similar across the three experimental groups.

## Discussion

The important new findings of our study are as follows. 1) Diabetic rats exhibit larger infarct volume and increased functional deficits than non-diabetic rats. 2) Anesthetic sevoflurane post-conditioning or mitoK_ATP_ channel opener diazoxide failed to confer neuroprotection in diabetic rats compared with non-diabetic rats. 3) Inhibition of mitoK_ATP_ channels blocked the neuroprotective effect of sevoflurane post-conditioning in non-diabetic rats, whereas it had no effect in diabetic rats. 3) Expression of mitoK_ATP_ channel subunit Kir6.2 in the brain was significantly reduced in diabetic rats as compared to non-diabetic rats. 4) Correction of hyperglycemia with insulin normalized expression of mitoK_ATP_ channel subunit Kir6.2 in the brain, prevented exacerbating ischemic brain injury and restored neuroprotective effects of sevoflurane post-conditioning in diabetes. Taken together, these results suggest that decreased brain mitoK_ATP_ channel by hyperglycemia contributes to exacerbating brain injury and the failure of sevoflurane post-conditioning to protect against ischemic brain injury in diabetes. Insulin glycemic control can prevents alteration in brain mitoK_ATP_ channel, consequently limit ischemic brain injury and restore neuroprotective effects of anesthetic post-conditioning in diabetes.

Diabetes is an important risk factor for cerebral ischemia, and the risk for ischemic stroke is about twice as much in a diabetic individual as in an non-diabetic individual [3,17]. In addition, brain injury is greater, and functional outcomes are worse following cerebral ischemia during diabetes [6,18]. In present study, we found that diabetic rats exhibited larger infarct volume and worse functional deficits compared with non-diabetic rats, whereas insulin-treated diabetic rats had reduced infarct volume and improved functional deficits. These results are consistent with earlier study [19] in which hyperglycemic and diabetes rats had similar increased infarct volume and functional deficits following cerebral ischemia as compared to control rats, insulin-treated diabetic rats exhibited an infarct volume and neurologic outcome similar to those observed in normoglycemic rats. These data suggested that elevated blood glucose concentrations are of major importance in the determination of the extent of brain injury. The mechanisms by which elevated blood glucose exacerbate ischemic brain injury remain poorly understood. MitoK_ATP_ channels have recently been demonstrated to involve in protective responses to ischemia in a variety of organs and tissues, including brain [9]. MitoK_ATP_ channel activity is regulated by intracellular ATP and ADP concentrations. An increased ratio of [ATP] to [ADP] results in decreased probability of K_ATP_ channel opening, whereas a decrease in this ratio promotes opening of these channels. K_ATP_ channel activation tends to hyperpolarize the cell by shifting the membrane potential toward the potassium equilibrium potential. Thus, K_ATP_ channels couple cell metabolism to cell membrane potential, thereby regulating many cellular activities [9]. K_ATP_ channels are hetero-octamers composed of pore-forming Kir6.x (6.1 or 6.2) subunits and sulfonylurea receptor (SUR1 or SUR2) regulatory subunits [9,20,21]. The mRNA transcripts of Kir6.2 and SUR1 were predominantly found in the brain [9,22]. *In vivo* experimental study using Kir6.2^−/−^ K_ATP_ channel knockout mice has shown that focal cerebral ischemia induced severe neurological deficits and large cortical infarcts in Kir6.2^−/−^ mice, but not in wild type mice [9]. *In vitro* experimental ischemia on cortical brain slices also revealed a substantial number of damaged cells in the Kir6.2^−/−^ group, but few degenerating neurons in the wild type group [9]. These *in vivo* and *in vitro* studies demonstrated that Kir6.2 channel in the brain plan an important role in protecting neurons against ischemia damage. Reduced Kir6.2 expression may lead to reduced K_ATP_ channel density, resulting in decreased K_ATP_ channel activity [23,24]. In current study, molecular studies revealed that both mRNA and protein levels of Kir6.2 in the brain was significantly decreased in diabetic rats 8 weeks after STZ injection as compared to non-diabetic rats. At this time point, middle cerebral artery occlusion induced increased brain injury. In addition, correction of hyperglycemia by insulin treatment in diabetic rats normalized expression of brain Kir6.2, consequently prevented the exacerbating brain injury. Our results indicated that decreased brain mitoK_ATP_ channel Kir6.2 is responsible for increased brain injury following cerebral ischemia in diabetes.

The underlying mechanisms for decreased expression of brain Kir6.2 channel in the present study remain to be determined. Diabetic rats 8 weeks after STZ infusion showed hyperglycemia and hypoinsulinemia, both might contribute to decreased expression of brain Kir6.2. However, numerous studies have suggested that hyperglycemia, but not hypo- or hyperinsulinemia, is associated with decreased expression of Kir6.2 channel. For example, Zucker diabetic fatty rats with hyperglycemia and hyperinsulinemia showed a 34% decrease in expression of Kir6.2 mRNA in pancreatic islets compared with lean control rats [25]. *In vivo* study investigating specific regulation of mitoK_ATP_ channel expression in the brain by peripheral metabolic signals demonstrated that hyperglycemia decreased Kir6.2 mRNA in the brain of rats in both the presence and absence of hyperinsulinemia [11,26]. In addition, cultured rat islets or the rat insulinoma cell line INS-I showed a significant decrease in Kir6.2 expression and activity when exposed to high glucose concentrations [27]. Conversely, glucose deprivation resulted in upregulation of the translation of existing Kir6.2 mRNA [28]. Thus, decreased expression of brain Kir6.2 channel is most likely due to hyperglycemia in the present study, although we did not monitor the time course of changes in expression of brain mitoK_ATP_ channels during development of diabetes.

Anesthetic pre- or post-conditioning is a powerful endogenous mechanism protecting brain and heart against ischemia injury. Influences of diabetes on cardioprotection induced by anesthetic pre- or post-conditioning have been extensively investigated in both clinical and experimental studies [29–31]. It is not known, however, whether increased ischemic brain injury observed in diabetes can be attenuated by these strategies. In present study, we found that post-conditioning with sevoflurane significantly reduced cerebral ischemia-reperfusion induced infarct volume and improved neurologic outcome in non-diabetic rats but not in diabetic rats. However, these beneficial effects were elicited in diabetic rats receiving a 4-week insulin treatment. Our study extended the previous results and demonstrated for the first time that diabetes inhibited sevoflurane post-conditioning induced neuroprotection and this protective strategy could be restored by insulin treatment.

It has been reported that anesthetic post-conditioning provided similar reductions in cerebral ischemia reperfusion damage when compared with anesthetic pre-conditioning group, and they may share common pathways [8]. Although numerous mechanisms are involved in protective effects of anesthetic pre- and post-conditioning, opening of mitoK_ATP_ channels is recently recognized to mediate neuroprotective actions of anesthetic pre- and post-conditioning [8]. Opening of the mitoK_ATP_ channels is associated with an uptake of potassium in the mitochondrial matrix which could maintain mitochondrial matrix volume by limitation of Ca^2+^ accumulation [8,32,33]. This reduction of mitochondrial Ca^2+^ accumulation would prevent, during reperfusion, the opening of the mitochondrial permeability transition pore known to inhibit oxidative phosphorylation and facilitate the release of proapoptotic proteins [8,34]. Opening of mitoK_ATP_ channels can also preserve endogenous antioxidant enzymes and attenuate oxidative stress in the myocardium during ischemia and reperfusion [35]. Our present results showed that inhibition of mitoK_ATP_ channels in non-diabetic rats with 5-HD abolished the protection conferred by sevoflurane post-conditioning, suggesting that under normal conditions, opening of mitoK_ATP_ channels is critical for sevoflurane post-conditioning, consistent with previous studies [8,10,36]. In contrast, 5-HD did not change infarct volume and functional deficits in diabetic rats after sevoflurane post-conditioning. In addition, diazoxide, which has been used extensively to study pharmacological pre-conditioning to confer cardioprotection or neuroprotection by activating mitoK_ATP_ channels [16,37], induced similar significant reduction in infarct volume and improvement in functional deficits in non-diabetic rats but not in diabetic rats, suggesting that brain mitoK_ATP_ channels were impaired in diabetic rats. Molecular studies clearly confirmed that diabetes induced alteration of mitoK_ATP_ channel in the brain as evidenced by decreased brain Kir6.2 expression. We speculate that decreased brain Kir6.2 expression results in reduced Kir6.2 activity, which might be insufficient to prevent Ca^2+^ accumulation during reperfusion in the response to anesthetic post-conditioning, resulting in the opening of the mitochondrial permeability transition pore which impairs oxidative phosphorylation and induces the release of proapoptotic proteins. Furthermore, elevated oxidative stress observed in the brain of diabetic animals [38,39] has been shown to contribute to increased ischemic brain injury [6]. Decreased brain Kir6.2 expression may lead to loss of ability to preserve endogenous antioxidant enzymes and attenuate oxidative stress in the brain of diabetic rats during ischemia and reperfusion, resulting in the failure of neuroprotection by sevoflurane post-conditioning. Indeed, impaired mitoK_ATP_ channels have been demonstrated to induce the inability to response to pre- and post-conditioning in diabetic human myocardium [40] or animal hearts [16,41].

Several limitations of the present study should be acknowledged. First, a rat model of STZ-induced diabetics was used in our study. This animal model represents human type 1 diabetes which is characterised by insulinopenia, hyperglycaemia and a largely unaffected lipid profile. In contrast, type 2 diabetes is characterized by a period of insulin resistance, hyperinsulinaemia and euglycaemia preceding the onset of hyperglycaemia. The differences in metabolic aberration and the onset of disease in the two types of diabetes may affect brain responsiveness to ischemia-reperfusion and anesthetic post-conditioning. Further studies are needed to examine the tolerance to ischemia-reperfusion and the ability of anesthetic post-conditioning to protect against ischemic brain injury in experimental models of type 2 diabetes, especially in a diet-induced type 2 diabetic animal model which is much closer to human type 2 diabetes presented in most stroke-patients. Second, the present study focused only on the role of impaired Kir6.2 channel in exacerbating ischemic brain injury and the failure of neuroprotection by anesthetic post-conditioning in diabetes, it is notable that correction of hyperglycemia with insulin normalized expression of brain Kir6.2 but did not completely restore the increased brain injury and the failure of neuroprotection by sevoflurane post-conditioning observed in diabetic rats to that observed in nondiabetic rats, suggesting that impaired Kir6.2 channel is not the only mechanism for exacerbating ischemic brain injury in diabetes. Finally, the present study demonstrated that long-term insulin treatment attenuated exacerbating ischemic brain injury and restored neuroprotective effects of anesthetic post-conditioning in diabetic rats, but we could not exclude the possibility that short-term insulin treatment might have neuroprotective effects independent of Kir6.2 expression. However, recent study reported that short-term insulin treatment (48 hours) normalized blood glucose concentration but neither reduced myocardial infarct size nor restored the cardioprotective effects of sevoflurane post-conditioning in STZ-induced diabetic rats [31]. Further studies are needed to determine whether organ***-***specific responses to short-term insulin treatment exist between heart and brain.

In conclusion, the present study demonstrates that diabetes exacerbate ischemic brain injury and abrogates the ability of sevoflurane post-conditioning induced neuroprotection, possibly due to decreased mitoK_ATP_ channel in the brain. Insulin glycemic control in diabetes may modulate brain mitoK_ATP_ channel, thus reducing the brain injury and restoring neuroprotection elicited by sevoflurane post-conditioning. Targeting brain mitoK_ATP_ channels may be critical to reduce ischemic brain injury and elicit neuroprotection by anesthetic post-conditioning in human diabetes.
